# Risk Factors for Tube Shunt Exposure: A Matched Case-Control Study

**DOI:** 10.1155/2013/196215

**Published:** 2013-07-21

**Authors:** Michael S. Koval, Fouad F. El Sayyad, Nicholas P. Bell, Alice Z. Chuang, David A. Lee, Stephen M. Hypes, Davinder S. Grover, Laura A. Baker, Stephen M. Huddleston, Donald L. Budenz, Robert M. Feldman

**Affiliations:** ^1^Ruiz Department of Ophthalmology and Visual Science, The University of Texas Medical School at Houston, 6431 Fannin Street, MSB 7.024, Houston, TX 77030, USA; ^2^Glaucoma Service, Wills Eye Institute, 840 Walnut Street, Philadelphia, PA 19107, USA; ^3^Bascom Palmer Eye Institute, Department of Ophthalmology, Miller School of Medicine, University of Miami, 900 N.W. 17th Street, Miami, FL 33136, USA; ^4^Robert Cizik Eye Clinic, 6400 Fannin Street, Suite 1800, Houston, TX 77030, USA; ^5^West Virginia School of Osteopathic Medicine, 400 North Lee Street, Lewisburg, WV 24901, USA; ^6^Glaucoma Associates of Texas, 10740 N. Central Expressway, Suite 300, Dallas, TX 75231, USA; ^7^Department of Ophthalmology, The University of Tennessee Health Science Center, Hamilton Eye Institute, 920 Madison Avenue, Memphis, TN 38103, USA; ^8^Department of Ophthalmology, University of North Carolina, 5151 Bioinformatics Building, CB 7040, Chapel Hill, NC 27599, USA

## Abstract

*Purpose*. To evaluate potential risk factors for developing tube shunt exposure in glaucoma patients. *Patients and Methods*. Forty-one cases from 41 patients that had tube shunt exposure from 1996 to 2005 were identified from the Robert Cizik Eye Clinic and Bascom Palmer Eye Institute. Each case was matched with 2 controls of the same gender and with tube shunts implanted within 6 months of the index case. Conditional logistic regression was used to determine risk factors. *Results*. The study cohort includes a total of 121 eyes from 121 patients. The mean age was 63.6 ± 19.7 years, ranging from 1 to 96 years. The average time to exposure was 19.29 ± 23.75 months (range 0.36–85.74 months). Risk factors associated with tube exposure were Hispanic ethnicity (*P* = 0.0115; OR = 3.6; 95% CI, 1.3–9.7), neovascular glaucoma (*P* = 0.0064; OR = 28.5; 95% CI, 2.6–316.9), previous trabeculectomy (*P* = 0.0070; OR = 5.3; 95% CI, 1.6–17.7), and combined surgery (*P* = 0.0381; OR = 3.7; 95% CI, 1.1–12.7). *Conclusions*. Hispanic ethnicity, neovascular glaucoma, previous trabeculectomy, and combined surgery were identified as potential risk factors for tube shunt exposure. These potential risk factors should be considered when determining the indication for performing tube shunt implantation and the frequency of long-term followup.

## 1. Introduction

Tube shunts have historically been used to treat glaucoma in cases that are refractory to filtering surgery or in those where filtering surgery is unlikely to be successful. In recent years, the indications have broadened, with some clinicians advocating shunts as primary surgical treatment for advanced glaucoma [1]. The overall use of tube shunts has been steadily increasing over the past decade, with one study showing an annual increase in the number of shunts placed between the years of 1995 and 2004 totaling 184% [2]. These devices function by draining aqueous through a silicone tube to a reservoir plate, which is covered by Tenon's capsule and conjunctiva. The tube is generally covered by one of a variety of patch materials where it enters the eye to prevent exposure through the overlying tissue. Such exposures may lead to more serious complications, such as endophthalmitis, if not promptly identified and treated [3].

The Tube versus Trabeculectomy and Ahmed Baerveldt Comparison studies reported the incidence of tube exposure as 5% at 5 years of followup and 1% at 1 year of followup, respectively [4, 5]. The specific causes of tube exposure, however, have not been clearly elucidated in the literature. Byun et al. concluded that the number of previous ocular surgeries may be a risk factor for tube exposure [6]. Although Huddleston et al. examined risk factors for failure of repair of tube shunt exposures [7], we are unaware of previous reports in the literature evaluating risk factors for developing tube shunt exposures. 

The purpose of this study was to evaluate potential risk factors for developing tube shunt exposures in glaucoma patients. 

## 2. Patients and Methods

A retrospective matched case-control study was conducted by reviewing charts from the Robert Cizik Eye Clinic at the Ruiz Department of Ophthalmology and Visual Science at The University of Texas Medical School in Houston (UTH) and the Bascom Palmer Eye Institute (BPEI) at the Miller School of Medicine, University of Miami. The Institutional Review Boards (IRBs) from each institution determined that this study was exempt for IRB review prior to initiation. Patient information was collected from charts using uniform data collection sheets in full compliance with HIPPA regulations. All research methods were in compliance with the Declaration of Helsinki and all federal and state laws of the United States.

Potential cases were identified using a computerized search of current procedural terminology (CPT) codes for repair of glaucoma drainage device between the years of 1996 and 2005 and were included in the study if the reason for repair was tube shunt exposure. Two controls were matched to each case based on implantation of a glaucoma drainage device within 6 months of implantation of the corresponding case. Controls were also matched to cases based on gender.

Data collected included demographics (age, race/ethnicity (self-reported), and gender) and presence of comorbid conditions (diabetes, hypertension, and history of autoimmune diseases). Baseline ocular information collected included the type of glaucoma, number of intraocular-pressure- (IOP-) lowering medications at the visit prior to initial implantation of tube shunt, number and type of prior glaucoma surgeries, and IOP. Number and type of previous ocular surgeries were also collected. Details of the technique of tube shunt implantation were recorded, including type of drainage device, device location, tube entry site, patch graft material, and whether or not it was combined with another surgical intervention. Date of diagnosis of exposure was also recorded. Data collected from each institution were combined into a unified data set. 

Descriptive statistics, mean and standard deviation, were calculated for continuous variables, that is, age, IOP, and so forth, and frequency and percentage were calculated for discrete variables, that is, gender, race, primary diagnosis, and so forth. The two-sample *t*-test or the Fisher exact test was used to compare groups. A stepwise conditional logistic regression was used to identify risk factors associated with tube exposure through the conjunctiva. The model contained an outcome variable, tube exposure (yes/no), and risk factors, including center (UTH versus BPEI), age, ethnicity (Hispanic versus non-Hispanic), history of diabetes mellitus, history of hypertension, history of autoimmune disease, type of glaucoma (neovascular (NVG) versus others), number of topical glaucoma medications used before shunt implantation, number of previous ocular surgeries (3 or more versus less than 3), previous tube implantation, previous trabeculectomy surgery, previous glaucoma laser surgery, previous retinal surgery, previous vitrectomy, shunt implantation combined with other surgery, and tube location. A risk factor was selected if the *P* value was less than 0.15 and was removed if *P* value > 0.05. 

The analyses were performed using SAS v9.2 (SAS Institute, Inc., Cary, NC). *P* values < 0.05 were considered statistically significant. *P* values were obtained from the two-sample *t-*test for continuous variables, Fisher exact test for discrete variables, and from the stepwise conditional logistic regression for risk factors. *P* values < 0.05 were considered statistically significant.

## 3. Results

A total of 41 cases from 41 patients were identified, 17 from UTH and 24 from BPEI. Each case was matched with 2 controls. Two cases from BPEI were only matched with one control due to lack of appropriate matches within the specified time frame. Thus, the study cohort included a total of 121 eyes from 121 patients, 51 eyes from UTH and 70 from BPEI. Thirty-eight patients (31.4%) were black, 37 (30.6%) were Hispanic, 44 (36.4%) were white, and 2 were Asian. Seventy-three (60.3%) were women. The mean age was 63.6 ± 19.7 years, ranging from 1 to 96 years. Thirty patients (24.8%) had diabetes mellitus, and 48 (39.7%) had hypertension. The average time to exposure was 19.29 ± 23.75 months (range 0.36–85.74 months). 

For the case group, the time to exposure was bimodally distributed with an average of 19.29 ± 23.75 months (range 0.36–85.74 months), while in the control group, the follow-up duration was approximately normally distributed with an average of 56.60 ± 38.02 months (range 0.303–161.1 months). In the exposure (cases) group, 33 patients (80%) had tube exposures, while 7 (17%) had plate exposures; one was unknown.

Data were evaluated on the potential risk factors as described earlier in Section 2. There were not enough cases/controls to evaluate the effect of autoimmune diseases (1 case, 2 controls) or inferior versus superior location.  Table 1 summarizes demographics and preoperative characteristics before undergoing initial tube shunt implantation and tube shunt implantation parameters for each group. The Fisher exact test found statistically significant differences in race/ethnicity (*P* = 0.015), type of glaucoma (*P* = 0.0235), prior panretinal photocoagulation (PRP; *P* = 0.0002), and 3 or more ocular surgeries prior to the tube implantation (*P* = 0.0014). Of the 15 NVG eyes, 7 had PRP procedures prior to shunt implantation; 5 of these had shunt exposure later. The percentage of eyes having 3 or more surgical procedures done before shunt implantation was significantly higher in cases than in controls (58.8% cases versus 27.0% controls; *P* = 0.0014).

Using a stepwise conditional logistic regression, risk factors associated with tube exposure were Hispanic ethnicity (*P* = 0.0115; odds ratio (OR) = 3.6; 95% confidence interval (CI), 1.3–9.7), NVG (*P* = 0.0064; OR = 28.5; 95% CI, 2.6–316.9), previous trabeculectomy (*P* = 0.0070; OR = 5.3; 95% CI, 1.6–17.7), and combined surgery (*P* = 0.0381; OR = 3.7; 95% CI, 1.1–12.7). Hispanics eyes experienced 3.6 times higher risk of tube exposure than non-Hispanics; NVG eyes experienced nearly 30 times higher risk than other types of glaucoma eyes. Eyes that had previous trabeculectomy increased the risk of exposure about 5 times, and combined tube implantation surgeries with other surgeries also increased the risk of exposure about 4 times (Table 2).

## 4. Discussion

 Tube shunt exposure is an infrequent complication, which may explain the paucity of the literature on the subject. To the best of our knowledge, this is the first controlled study evaluating potential risk factors for tube shunt exposure. The vast majority of tube shunts were Baerveldt Glaucoma Implants (Abbott Medical Optics, Inc., Santa Ana, CA) in this study (most likely due to surgeon preference or patient necessity), and there has been no study evaluating risk factors for this complication in Baerveldt shunts. There are, however, a few proposed theories on the etiology of tube shunt exposure. Lankaranian et al. suggested that the mechanical forces of the eyelid as well as the pressure of the underlying tube may contribute to conjunctival exposures [1, 8], while Smith et al. proposed both mechanical and immunologic factors contributing [9]. The intention of the current study was to examine the potential factors which might predispose to exposure based on the previous theories. Unfortunately, our numbers of patients with autoimmune diseases and inferiorly placed shunts were few, and there was not adequate power to evaluate their impact.

 In the only similar study by Byun et al., Ahmed Glaucoma Valves (New World Medical, Inc., Rancho Cucamonga, CA) were examined in a total of 11 cases of conjunctival exposure. The authors of this study concluded that the only risk factor for exposure was the number of previous ocular surgeries. They concluded that NVG was not a risk factor for exposure, and ethnicity was not a variable that was considered, as all of the patients appear to have been of Korean descent [6].

 In our study, Hispanic patients had a higher incidence of tube exposure than non-Hispanics [7]. It is unclear why Hispanics would have a higher incidence of exposures than non-Hispanics; however, it is possible that there are socioeconomic factors that cannot be elucidated by the data collected in this retrospective study. The Hispanics in the 2 locations are also potentially ancestrally distinct. Elucidating the finding that Hispanics were at higher risk of tube exposure was likely only possible because both sites have a high proportion of patients who are ethnically Hispanic [10].

 Neovascular glaucoma (NVG) was also found to be a risk factor for tube shunt exposure. The most common cause of NVG in this population was diabetes mellitus, and a large proportion of the patients in this study with NVG also had diabetic retinopathy (8 of the 15 with NVG). Diabetics are known to have vascular changes in the conjunctiva similar to those found in the retina, suggesting that there is poor conjunctival perfusion [11], which may theoretically result in less tissue modulation and poorer tissue strength. Prior PRP was more common in cases than controls in this study, with 15 (36.6%) cases and only 6 (7.7%) controls having had prior treatment with PRP. However, this effect did not appear in the multivariate analysis. This suggests that the relationship between PRP and tube shunt exposures is more likely a relationship between the underlying disease (NVG), which required the PRP, and tube shunt exposure than the PRP itself.

 As there is an elevated rate of diabetes in Hispanic populations [10] and with neovascular glaucoma also found to be a risk factor in this study, there may be a potential relationship to be elucidated between these 3 factors. However, there were only 8 patients with diabetes and neovascular glaucoma and only 2 of those were Hispanic (both in case group; none in control group); these small numbers make it difficult to draw conclusions on this relationship. 

Prior trabeculectomy was found to be a risk factor for tube shunt exposure. The data collected for this study came from patients with tube shunt implantation between the years of 1996 and 2005, a time when mitomycin-C and 5-fluorouracil were frequently used to modulate wound healing in trabeculectomies at both BPEI and UTH, as well as many other clinical practices. The long-term results of aggressive use of these antifibrotic agents have been to increase the numbers of bleb breakdowns and wound leaks [12]. A similar process that resulted in ischemic blebs and bleb breakdown could result in late tube shunt exposures. This is likely a true risk factor because the interaction effect of previous trabeculectomies and multiple previous surgeries was not significant (*P* = 0.1916) in the conditional logistic regression. The relationship between antifibrotic regimen used in trabeculectomy and later tube shunt exposure cannot be evaluated due to a lack of trabeculectomies being performed during that time frame at these 2 institutions without the use of these agents. Thus the health of the conjunctiva after trabeculectomy may be an important factor in the prevention of tube exposures.

 Another risk factor for tube shunt exposure was tube shunt implantation combined with another surgery, most often cataract surgery or vitrectomy. Cataract surgery is commonly performed in combination with tube shunt implantation because of the possibility of progression of cataract after tube shunt implantation in a phakic eye [13]. It is possible that the technique of tube shunt positioning was altered to be combined with another surgery. The operative reports were not useful to delineate such a difference. Combining tube shunt implantation with vitrectomy is generally performed in complicated cases with poor underlying tissue health, such as in neovascular eyes, uveitic eyes, or in cases of intended pars plana placement (i.e., eyes with preexisting penetrating keratoplasty). Thus, implant positioning alone may not be solely responsible for this increased risk.

Based on the results of this study, it appears that a number of different risk factors may increase the risk of tube shunt exposure. Co-morbid medical conditions, such as diabetes, that lead to ischemia of ocular tissues appear to be an important factor to consider in patients with this surgery. The ocular surgical history also plays a significant role in the ability to successfully cover the tube. One weakness of this study is the retrospective nature of data collection, which prevents useful evaluation of potential risk factors which are not available in the data set. 

The majority of tube shunts in this study were of the Baerveldt variety (97.6% and 96.3% in cases and controls, resp.), which limited the ability of this study to evaluate the different types of tube shunts. The majority of patch graft material used in both cases and controls was sclera (70.7% and 62.5%, resp.), which also limited the ability of the study to compare the different types of patch graft material in a similar way. Although we intended to look at graft type as a potential risk factor, the study was underpowered to determine any useful finding.

In conclusion, this study identified Hispanic ethnicity, neovascular glaucoma, prior trabeculectomy, and combined surgery as potential risk factors for tube shunt exposure. These risk factors should be considered when determining the indication for performing tube shunt implantation and the frequency of long-term followup.

## Figures and Tables

**Table 1:** Demographics/preoperative glaucoma patient characteristics and tube shunt implantation parameters.

Variable	Cases	Controls	*P* value^a^
Center, no. of cases/controls (%)			1.000
BPEI	24 (58.5)	46 (57.5)	
UTH	17 (41.5)	34 (42.5)	

Demographics			
Sex (female, %)^b^	25 (61.0)	48 (60.0)	1.000
Race/ethnicity, no. of patients (%)			0.015^c^
White	12 (29.3)	32 (40.0)	
Black	9 (22.0)	29 (36.3)	
Hispanic	20 (48.8)	17 (21.3)	
Asian	0 (0.0)	2 (2.5)	
Age, mean years (SD)	64.2 (19.1)	63.6 (20.2)	0.8713

Comorbid systemic diseases			
Diabetes, no. of eyes (%)	12 (29.3)	18 (22.5)	0.5054
Hypertension, no. of eyes (%)	17 (41.5)	31 (38.8)	0.8452

Baseline ocular information (before shunt implantation)			
Type of glaucoma, no. of eyes (%)			0.0235^c^
Primary open angle glaucoma	11 (26.8)	38 (47.5)	
Neovascular glaucoma (NVG)	9 (22.0)	6 (7.5)	
Others	21 (51.2)	36 (45.0)	
Preoperative intraocular pressure, mean mmHg (SD)	30.8 (11.2)	29.9 (10.1)	0.6573
No. of IOP-lowering medications, mean (SD)	2.1 (1.0)	2.3 (1.1)	0.4369
Previous trabeculectomy, no. of eyes (%)	21 (51.2)	26 (32.9)^d^	0.0753
Previous tube shunt implantation, no. of eyes (%)	4 (9.8)	3 (3.3)	0.2285
Previous laser procedure (ALT), no. of eyes (%)	3 (7.3)	6 (7.7)^e^	1.000
Previous PK/PKP, no. of eyes (%)	4 (9.8)	6 (7.5)	0.7324
Previous PRP/EL, no. of eyes (%)	15 (36.6)	6 (7.7)^e^	0.0002^c^
Previous vitrectomy, no. of eyes (%)	9 (22.0)	9 (11.3)	0.1755
Previous ocular surgeries, no. of eyes 3 or more (%)	24 (58.5)	22 (27.5)	0.0014^c^

Tube shunt implantation parameters			
Type of shunt device, no. of Baerveldt (%)	40 (97.6)	77 (96.3)	1.000
Device location, no. of superotemporal (%)	40 (97.6)	73 (91.3)	0.2633
Tube entry site, no. of anterior chambers (%)	28 (92.7)	74 (92.5)	1.000
Type of patch graft used, no. of scleral patch grafts (%)	29 (70.7)	26 (62.5)	0.3416
Combined surgery, no. of eyes (%)	16 (39.0)	19 (19.8)	0.0928
Combined with cataract, no. of eyes (%)	6 (14.6)	12 (15.0)	1.000
Combined with PKP, no. of eyes (%)	2 (4.9)	1 (1.3)	0.2648
Combined with vitrectomy, no. of eyes (%)	9 (13.0)	10 (12.5)	0.1945

BPEI: Bascom Palmer Eye Institute; UTH: The University of Texas Medical School at Houston; NVG: neovascular glaucoma; PKP: penetrating keratoplasty; ALT: argon laser trabeculoplasty; PK: penetrating keratectomy; PRP: panretinal photocoagulation; EL: endolaser; SD: standard deviation; No.: number.

^a^Obtained from two-sample *t*-test for continuous variables and Fisher exact test for discrete variables.

^b^Sex was used to match cases to controls.

^c^Statistically significant, *P* < 0.05.

^d^One missing observation.

^e^Two missing observations.

Note: 5 eyes combined with both cataract and vitrectomy (1 case, 4 controls).

**Table 2:** Risk factors associated with tube exposure.

Risk factor	*β* ± SE	Odds ratio	*P* value	95% confidence interval
Hispanic ethnicity	1.28 ± 0.51	3.6	0.0115	1.3–9.7
Neovascular glaucoma	3.35 ± 1.23	28.5	0.0064	2.6–316.9
Previous trabeculectomy	1.66 ± 0.62	5.3	0.0070	1.6–17.7
Combined surgery	1.31 ± 0.63	3.7	0.0381	1.1–12.7

*β* ± SE: estimated parameter from the conditional logistic regression model with standard error.
